# Quantitative phosphoproteome on the silkworm (*Bombyx mori*) cells infected with baculovirus

**DOI:** 10.1186/s12985-017-0783-8

**Published:** 2017-06-19

**Authors:** Jauharotus Shobahah, Shengjie Xue, Dongbing Hu, Cui Zhao, Ming Wei, Yanping Quan, Wei Yu

**Affiliations:** 1 Institute of Biochemistry, College of Life Sciences, Zhejiang Sci-Tech University, Xiasha High-Tech Zone No.2 Road, Zhejiang Province Hangzhou, 310018 People’s Republic of China; 2 Zhejiang Provincial Key Laboratory of Silkworm Bioreactor and Biomedicine, Zhejiang Province Hangzhou, 310018 People’s Republic of China

**Keywords:** *Bombyx mori*, BmNPV, Phosphorylation, Proteomic, Tandem mass tag, Regulation

## Abstract

**Background:**

*Bombyx mori* has become an important model organism for many fundamental studies. *Bombyx mori* nucleopolyhedrovirus (BmNPV) is a significant pathogen to *Bombyx mori*, yet also an efficient vector for recombinant protein production. A previous study indicated that acetylation plays many vital roles in several cellular processes of *Bombyx mori* while global phosphorylation pattern upon BmNPV infection remains elusive.

**Method:**

Employing tandem mass tag (TMT) labeling and phosphorylation affinity enrichment followed by high-resolution LC-MS/MS analysis and intensive bioinformatics analysis, the quantitative phosphoproteome in *Bombyx mori* cells infected by BmNPV at 24 hpi with an MOI of 10 was extensively examined.

**Results:**

Totally, 6480 phosphorylation sites in 2112 protein groups were identified, among which 4764 sites in 1717 proteins were quantified. Among the quantified proteins, 81 up-regulated and 25 down-regulated sites were identified with significant criteria (the quantitative ratio above 1.3 was considered as up-regulation and below 0.77 was considered as down-regulation) and with significant *p*-value (*p* < 0.05). Some proteins of BmNPV were also hyperphosphorylated during infection, such as P6.9, 39 K, LEF-6, Ac58-like protein, Ac82-like protein and BRO-D.

**Conclusion:**

The phosphorylated proteins were primary involved in several specific functions, out of which, we focused on the binding activity, protein synthesis, viral replication and apoptosis through kinase activity.

## Background

*Bombyx mori*, the domesticated silkworm, is an economically significant insect in silk production [1]. Farmers in many developing countries such as China, India, Brazil, Vietnam, and Thailand are evolving sericulture as a foremost income source. China, the biggest cocoon producer (almost 80%) worldwide, generated a sericulture-based income of 22.4 billion ¥ (Yuan) (approximately equal to USD 3.24 billion by March 2017) by producing 6.61 × 10^8^ kg of cocoons in 2011 [2]. Moreover, this lepidopteran insect has become a model organism for several fundamental studies in biochemistry, molecular genetics, and genomics [3, 4]. In-depth studies of silkworm were extensively carried out since its genome was completely sequenced in 2004 [1]. H3 N-terminus, an N-terminal tail of histone H3 in holocentric chromosomes of the silkworm, is reported to be extensively acetylated and methylated, which is almost similar to that of mammals [5]. Furthermore, the proteome of *B. mori* was detected to harbor a total of 342 acetylated proteins with 667 Kac (Lysine acetylation) sites [6]. However, some pathogenic agents such as viruses, fungi, and bacteria pose serious biological challenges for this sericulture causing almost 20% losses of the potential cocoon production each year. *Bombyx mori* nucleopolyhedrovirus (BmNPV), a member of baculoviridae family, is the pivotal viral pathogen of *B. mori* that could elicit almost 80% economic loses in sericultural industry [7]. On the other hand, BmNPV has been used as an effective, feasible, and safe technology for the production of recombinant proteins in insect or insect cultured cells called BEVS (baculovirus expression vector system) [8]. This system produces a large amount of the desired proteins by infecting insect cells with a virus encoding the desired transgene under the robust baculovirus polyhedrin promoter [9].

Post-translational modification (PTM) is one of three levels of protein activity regulation that could efficiently drive adaptive cellular responses by adding or removing functional groups from protein residues [10]. In addition to acetylation and methylation as the variety of PTM, reversible phosphorylation is an important regulatory mechanism that occurs in both prokaryotic and eukaryotic organisms [11]. Reversible phosphorylation results in a conformational change affecting some regulatory processes such as biological thermodynamics of energy-requiring reactions, enzyme inhibition and activation, protein interaction via domain recognition, and protein degradation [12–14].

Major efforts have been invested to study the phosphorylation in several species including insects. It has been reported that BRO proteins of BmNPV undergo phosphorylation when the virus infects the host cells and reaches the maximum of phosphorylation level between 14 and 20 h p.i. which was proposed that it has effects on DNA and RNA binding activity regulations [15]. Although phosphorylation has been examined for its crucial role in the regulation of metabolism, the investigation on the protein for its phosphorylation in silkworm is yet lacking. Moreover, BmNPV is not just the most influential factor on *B. mori* but also useful vector on BEVS system*.* Hence, the study of the effect of infection on the global phosphoproteome pattern in *B. mori* is important to comprehend and discover advanced inventions in both fields. Employing TMT (tandem mass tag) labeling and phosphorylation affinity enrichment followed by high-resolution LC-MS/MS analysis, we analyzed the quantitative phosphoproteome in the pair of experimental conditions (control and infected by BmNPV) to elucidate the comprehensive profiling of phosphorylation on the proteome of *B. mori* after the infection of BmNPV. Altogether, 6480 phosphorylation sites in 2112 protein groups were identified, among which 4764 sites in 1717 proteins were quantified. Some proteins of BmNPV also underwent post-translational modification such as p6.9 and 39 K proteins which interestingly have 2 and 4 up-regulation sites, respectively. Thus, our results will provide a fundamental resource for the prospective study of the phosphorylation in *B. mori.*

## Methods

### Cell culture and viral infection

*B. mori* cells, originating from the ovary of silkworm, are maintained in our laboratory. Cells were grown at 27 °C in Sf-900 medium (Thermo Fisher Scientific, USA) complemented with 10% fetal bovine serum. The BmN cells were cultured at a density of 1 × 10^6^ cells in 25 cm^2^ flask. In the present study, two categories of cells were examined: control cells (normal cell) and those that were infected with the preserved virus, *B. mori* nucleopolyhedrovirus (BmNPV), in 24 h using 10 MOI (multiplicity of infection). The cell culture and viral infection experiments were done as biological triplicates. After infection, the cells were collected and centrifuged at 3000 rpm. The control cells were treated similarly. Both pellets were washed twice with PBS after the supernatant was discarded.

### Protein extraction

The samples were mildly sonicated three times on ice employing a high intensity ultrasonic processor (Scientz, Ningbo Scientz Biotechnology Co., Ltd) in lysis buffer (8 M urea, 2 mM EDTA, 10 mM dithiothreitol [DTT], 2% phosphatase inhibitor cocktail V). Then, the samples were centrifuged at 20000 *g* at 4 °Cfor 10 min to eliminate the remaining debris. The proteins were precipitated using cold 15% trichloroacetic acid (TCA) for 2 h at −20 °C. The supernatant was discarded after the proteins were pelleted by centrifugation at 4 °Cfor10 min. The remaining precipitate was washed three times with cold acetone. The protein extract was re-suspended in buffer (8 M urea, 100 mM NH_4_HCO_3_, pH 8.0). The 2-D Quant kit (GE health care) was used to estimate the protein concentration according to the manufacturer’s instructions.

### Trypsin digestion

The protein solution was treated with 10 mM DTT for 1 h at 37 °C to obtain a reduced form followed by alkylation using 20 mM iodoacetamide (IAA) for 45 min at room temperature in darkness. Subsequently, for trypsin digestion, the protein was diluted with 100 mM triethylammonium bicarbonate (TEAB) to urea concentration less than 2 M. For the first digestion, the protein was added to trypsin at 1:50 trypsin-to-protein mass ratio overnight and for the second digestion at 1: 100 trypsin-to-protein mass ratio for 4 h.

### TMT labeling

Following the trypsin digestion, the peptides then were desalted by Strata-X C18 SPE column (Phenomenex) and vacuum-dried. After reconstitution in 0.5 M TEAB, the peptides were labeled using the tandem mass tag (TMT) according to the manufacturer’s instructions (Thermo Scientific) of the 6-plex TMT kit. Concisely, one unit of TMT reagent (defined as the amount of reagent required to label 1 mg of protein) was liquefied and reconstituted in ACN. The peptide mixtures were incubated for 2 h at room temperature, pooled, desalted, and dried by vacuum centrifugation.

### HLPC fractionation

High pH reverse-phase HPLC with Agilent 300 Extend C18 column (5 μm particles, 4.6 mm ID, 250 mm length) was used to fractionate the peptide samples. The peptides were separated with a gradient of 2–60% acetonitrile in 10 mM ammonium bicarbonate pH 10 over a period of 80 min into 80 fractions. The peptides then were combined into 8 fractions and dried by vacuum centrifugation.

### Affinity enrichment

First, the peptide mixtures were incubated with IMAC microspheres suspension under vibration. The centrifugation precipitated the IMAC microspheres enriched with phosphopeptides. The IMAC microspheres were then washed with 50% CAN/6% TFA and 30% CAN/0.1% TFA, sequentially, in order to remove the nonspecifically absorbed peptides. The elution buffer containing 10% NH_4_OH was added to elute the enriched phosphopeptides from the IMAC microspheres with vibration. The supernatant containing phosphatides were collected and lyophilized for LC-MC/MS analysis.

### LC-MS/MS analysis

The peptide samples were thawed in solvent A (0.1% FA in 2% CAN) and directly loaded onto a reversed-phase pre-column (Acclaim PepMap 100, Thermo Scientific). A reversed-phase analytical column (Acclaim PepMap RSLC C18 column, 50 μm × 15 mm, 2 μm, 100 Å, Thermo Fisher Scientific) was used to separate the peptides with a linear gradient of 4–22% solvent B (0.1% FA in 98% ACN) for 50 min, 22–35% solvent B for 12 min, 35–85% solvent B for 4 min, and holding at 85% for the last 4 min at a constant flow rate of 300 nL/min on an EASY-nLC 1000 UPLC system. The peptides analysis was conducted using Q Exactive™ Plus hybrid Quadrupole-Orbitrap mass spectrometer (ThermoFisher Scientific). The peptides were subjected to NSI (nanospray ion) source followed by tandem mass spectrometry (MS/MS) in Q Exactive™ Plus coupled online to the UPLC. The intact peptides were detected in the orbitrap at a resolution of 70,000. The peptides were selected for MS/MS using the NCE setting as 28 and ion fragments were detected in the Orbitrap at a resolution of 17,500. The top 20 precursor ions above a threshold ion count of 5.0E3 were applied with a data-dependent procedure which alternated between 1 MS scan followed by 20 MS/MS scans in the MS survey scan with 15.0 s dynamic exclusion. The applied electrospray voltage was 2.0 kV. Overfilling of the Orbitrap was prevented by employing an automatic gain control (AGC), and 5E4 ions were accumulated for the generation of MS/MS spectra. The fixed first mass was set as 100 m/z, and the MS scan range was 350–1800 m/z.

### MS/MS data analysis

The MS/MS data were analyzed using MaxQuant with integrated Andromeda search engine (v.1.4.1.2). The tandem mass spectra was searched against *B. mori* database concatenated with reverse decoy database. The peptides were cleaved by trypsin/P enzyme which allowed up to 2 missing cleavages, 5 modifications per peptide, and 5 charges. The mass error was set to 10 ppm for precursor ions and 0.02 Da for the fragment ions. The oxidation of methionine, the phosphorylation on serine, threonine, or tyrosine, and acetylation on the N-terminal of the protein were determined as variable modifications while carbamidomethylation on cysteine was determined as fixed modification. The false discovery rate (FDR) thresholds for protein, peptide, and modification site were specified at 1%. The minimum peptide length was set at 7 and TMT-6plex was selected for quantification. All the other parameters in MaxQuant were configured to default values. The site localization probability was set as >0.5.

### Bioinformatics analysis

Gene Ontology (GO) annotation proteome was derived from the UniProt-GOA database [http://www.ebi.ac.uk/GOA/]. The identified protein ID was first converted to UniProt ID and then mapped to GO IDs. The InterProScan software was employed to annotated protein’s GO functional database based on the sequence alignment method in case some identified proteins were not annotated by UniProt-GOA database. The proteins were then classified by Gene Ontology annotation based on three categories: biological processes, cellular components, and molecular functions. The KEGG (Kyoto encyclopedia of genes and genomes) pathway annotation was performed using KEGG online service tools KAAS according to the annotated protein’s KEGG database description. The domain functional description annotation was conducted by InterProScan based on the protein sequence alignment method and the InterPro domain database.

A functional enrichment analysis was performed based on the three types of annotation (GO, KEEG pathway, and domain annotation). For each category, a two-tailed Fisher’s exact test was employed to test the enrichment or depletion of the differentially expressed protein against all the resulting protein clusters. Standard FDR control methods were used to carry out the correction of multiple hypothesis testing. Any category with a *p*-value <0.05 in any of the clusters was considered to be significant.

For predicting the subcellular localization, we used WLF PSORT, an updated version of PSORT/PSORT II for the prediction of eukaryotic sequences. Soft motif-x was used to carry out the analysis of sequences model comprised of the amino acids in specific positions of modifying 21-mers (6 amino acids upstream and downstream of the site) in all protein sequences.

## Results

### Quantification of phosphoproteome

**Fig. 1:**
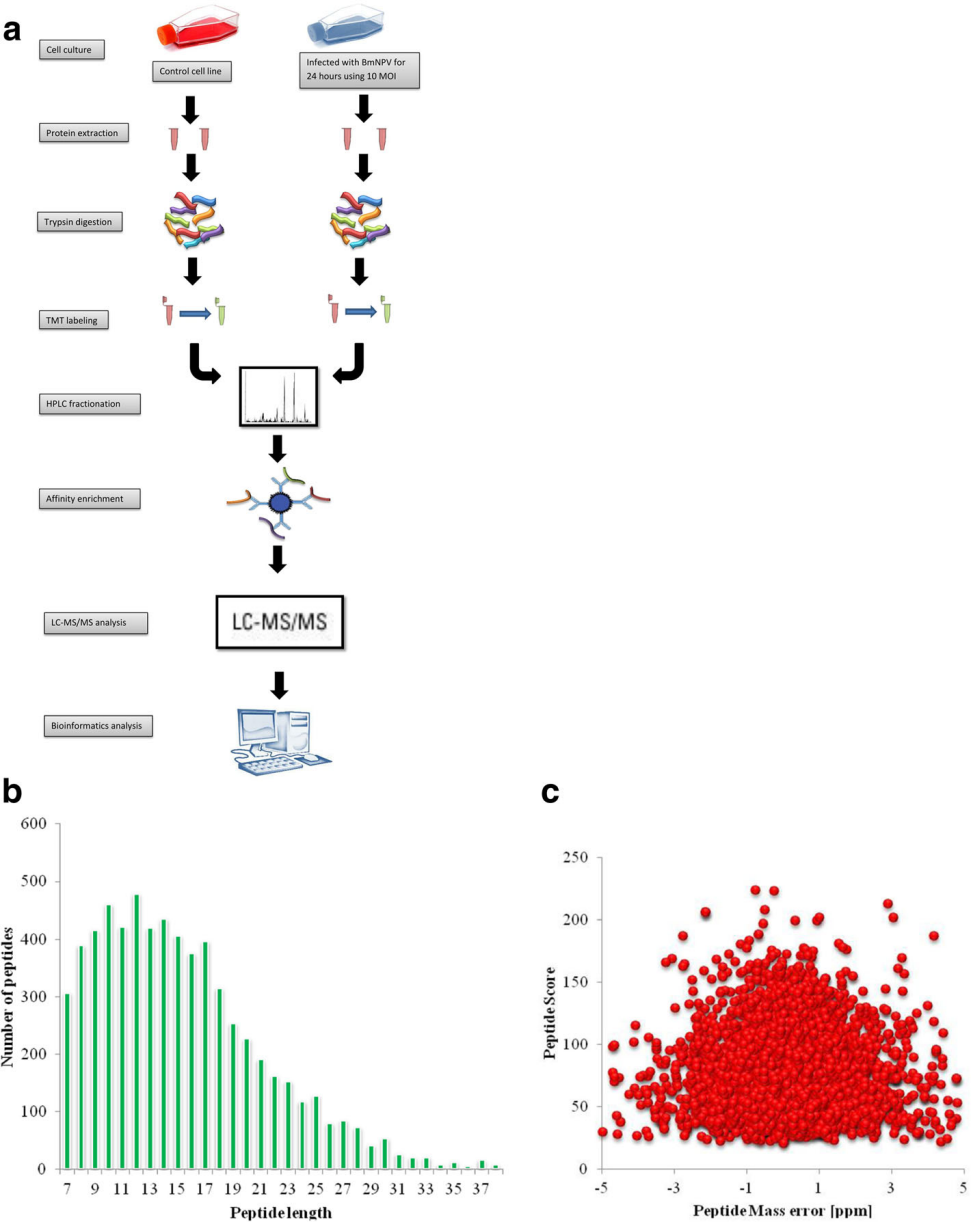
Schematic representation of the experimental design. **a** Schematic showing an overview of experimental design for the phosphoproteome analysis of *B. mori.* **b** The peptide length distribution of phosphorylated protein. C. Mass error of all identified peptides

Using TMT labeling and phosphorylation affinity enrichment followed by high-resolution LC-MS/MS analysis, we performed the very first quantitative phosphoproteome analysis aiming to compare the global pattern of phosphoproteome of *B. mori* after infection of BmNPV (Fig. 1a). Focusing on those phosphoproteins that were either up- or down-regulated after BmNPV infection, we identified 6480 phosphorylation sites in 2112 protein groups, among which 4764 sites in 1717 proteins were quantified. The fold-change cut-off was set when proteins with the quantitative ratios above 1.3 or below 0.77 were deemed to be significant. Among the quantified proteins, 81 hyperphosphorylated sites in 66 proteins and 25 hypophosphorylated sites in 24 proteins were identified after BmNPV infection. The distribution of peptides length from the prepared sample was between 8 and 20 (Fig. 1b), which was complied with the standard and in agreement with the property of tryptic peptides. The mass error of the peptide weight was almost zero, and most of them were less than 0.02 Da (Fig. 1c), indicating that the accuracy of MS data was as required.

Among the regulated phosphoproteins, some proteins possessed a remarkable elevation ratio, for example, origin recognition complex subunit 2 with the highest phosphorylation ratio 5.69 among the hyperphosphorylated proteins and uncharacterized protein LOC101739044 with the lowest phosphorylation ratio 0.37 among the hypophosphorylated proteins. Moreover, other proteins also occurred with more than one regulated site, i.e., thymosin isoform XI with 4up-regulated sites and upstream activation factor subunit spp27 with 2down-regulated sites (Table 1). There are also some notable proteins of BmNPV which were modified during infection. For instance, P6.9 has two up-regulation sites and 39 K protein has 4 up-regulation sites, one of which had a very high phosphorylation ratio of 16.68 fold. Other modulated BmNPV proteins also have more than one hyperphosphorylated site such as LEF-6, Ac58-like protein, Ac82-like protein, and BRO-D in most of which a relative high phosphorylation ratio was observed (Table 2). Those proteins with the recognizable characteristic either with the remarkable phosphorylation ratio or more than one regulated sites are strongly suggested to have a significant role in the alteration of several biological functions in silkworm cells after BmNPV infection.

**Table 1:** A partial list of phosphorylated protein of *B. mori* upon BmNPV infection

Protein accession	24/N Ratio	Regulated	24/N *P* value	Site	Protein names	Modified sequence
Binding						
H9JP10	2.31	Up	0.0100989	T	a ATP-dependent RNA helicase DDX10	_DDDGAET(ph)DQS(ph)IEENPEGK_
H9JMN2	1.50	Up	0.0423116	S	uncharacterized protein LOC101745112	_SSIANNKPLNISLKS(ph)PAK_
H9JIW2	1.35	Up	0.0289589	T	host cell factor 1	_VTPGT(ph)PTTK_
H9ITJ4	1.46	Up	0.0384895	S	ankyrin repeat domain-containing protein SOWAHC-like isoform X4	_IGS(ph)LNVR_
H9J723	1.31	Up	0.0051127	S	26S proteasome non-ATPase regulatory subunit 2 isoform X1	_HYETDNEQS(ph)STEDTAFK_
H9JQ06	1.39	Up	0.0448089	S	probable serine/threonine-protein kinase kinX	_NNPKPTS(ph)PPSSSK_
H9JFT9	1.45	Up	0.0137186	T	uncharacterized protein LOC101735968	_VKPLNIMDVELPNT(ph)QEIK_
H9JVR1	1.33	Up	0.0197819	S	protein Peter pan	_AGLLS(ph)ES(ph)EFEDDPNSQIVLPQSLASR_
H9JQ10	1.44	Up	0.0117537	S	DNA topoisomerase 2	_KPAEMDS(ph)DCLFDS(ph)LIEDAK_
H9JPZ9	1.43	Up	0.0157487	S	zinc finger CCCH domain-containing protein 15 homolog	_KNEIS(ph)LVDLIER_
H9IWZ6	1.46	Up	0.0249602	S	zinc finger protein 91-like isoform X2	_EIELTES(ph)DIEAER_
H9J889	1.31	Up	0.0092107	S	catenin delta-2 isoform X1	_SAS(ph)PGALPQHNLHLQQR_
H9J5A7	1.30	Up	0.0284892	S	afadin	_NLAQSQSNYGS(ph)GSIYDR_
H9JPQ3	1.59	Up	0.0416121	S	chaperonin	_SADELLNFS(ph)KGEESLLEK_
H9IZ67	2.61	Up	0.0391567	T	E3 ubiquitin-protein ligase listerin	_S(ph)PTRPDDT(ph)EDEQPTK_
H9JSA2	1.73	Up	0.0268143	S	protein fem-1 homolog CG6966 isoform X2	_NEDS(ph)FDFFGMMEK_
H9JP80	1.99	Up	0.0003232	S	thymosin isoform X1	_FDS(ph)SQLK_
H9JP80	2.01	Up	0.0189944	T	thymosin isoform X1	_SQLEGFNT(ph)SCLR_
H9JP80	2.91	Up	0.0175862	S	thymosin isoform X1	_SQLEGFNTS(ph)CLR_
H9JP80	1.83	Up	0.0118707	S	thymosin isoform X1	_FDSS(ph)QLK_
H9JDB6	1.48	Up	0.0205499	S	E3 SUMO-protein ligase RanBP2-like	_DEVVDPKPEAVFQVRPS(ph)PSK_
H9ISA9	1.34	Up	0.0038934	T	MAP/microtubule affinity-regulating kinase 3-like isoform X3	_QNT(ph)IDSASIK_
H9JD76	2.02	Up	0.0310478	S	tudor and KH domain-containing protein	_S(ph)LASGLEK_
H9JKS8	1.74	Up	0.0391086	S	ribonuclease L inhibitor homolog isoform X1	_TES(ph)LVFK_
H9J713	1.55	Up	0.0355302	S	putative uncharacterized protein DDB_G0282133	_ETQEQAYDS(ph)NDDSHSDSDIHR_
H9J3L2	1.36	Up	0.0256814	S	uncharacterized protein LOC101735988	_NYLTYKPNSES(ph)EFEDEK_
H9J0I3	1.43	Up	0.0219766	S	uncharacterized protein LOC101745835 isoform X3	_ALLHSFS(ph)TNDAALGEYK_
H9JKC7	1.54	Up	0.0383008	S	nucleolar and coiled-body phosphoprotein 1	_KEES(ph)S(ph)DDDS(ph)S(ph)DDESEK_
H9JKC7	1.31	Up	0.0280141	T	nucleolar and coiled-body phosphoprotein 1	_SVVT(ph)PAK_
H9JMB7	0.74	Down	0.0422832	S	pre-mRNA-processing factor 40 homolog A isoform X1	_SHS(ph)ASLSR_
H9JKK4	0.67	Down	0.0465377	S	gamma-taxilin	_HRLS(ph)QIQDQLK_
H9IXT6	0.72	Down	0.0486996	Y	tyrosine-protein kinase Abl isoform X1	_DDTY(ph)TAHAGAK_
H9IWP3	0.72	Down	0.0174047	S	uncharacterized protein LOC101739668 isoform X2	_ASVS(ph)S(ph)ASTTACDK_
H9J270	0.72	Down	0.0174573	S	serine/threonine-protein kinase OSR1	_RQPGAS(ph)GR_
H9JXK2	0.66	Down	0.0083349	S	forkhead box protein J2-like	_HS(ph)PLGPIPR_
H9JMK3	0.57	Down	0.0170053	S	upstream activation factor subunit spp27	_S(ph)ADDS(ph)DDDNWGKPTR_
H9JMK3	0.73	Down	0.0495388	S	upstream activation factor subunit spp27	_S(ph)ADDS(ph)DDDNWGKPTR_
H9J557	0.75	Down	0.0095049	S	brefeldin A-inhibited guanine nucleotide-exchange protein 1	_EETDHHS(ph)IK_
H9IWN8	0.77	Down	0.0145766	S	cytoplasmic FMR1-interacting protein	_QLS(ph)ADDAAGVEHVR_
H9J1U3	0.60	Down	0.0419989	T	serrate RNA effector molecule homolog isoform X2	_YNST(ph)DETEEKPAEKPAEK_
H9JNW9	0.70	Down	0.0104359	S	acetyl-CoA carboxylase isoform X2	_RGS(ph)IDDVLVK_
H9J3S7	0.71	Down	0.0174179	T	transcription factor jun-D	_LHHGEAVT(ph)PLGR_
protein synthesis						
H9JP10	2.31	Up	0.0100989	T	a ATP-dependent RNA helicase DDX10	_DDDGAET(ph)DQS(ph)IEENPEGK_
Q9BPS1	1.69	Up	0.0195419	S	Elongation factor 1delta	_SS(ph)LASEVAK_
H9J8J6	0.76	Down	0.0221005	S	U4/U6.U5 tri-snRNP-associated protein 1 isoform X1	_S(ph)RS(ph)PLEGEER_
replication						
H9JSI5	5.70	Up	0.0311772	S	origin recognition complex subunit 2	_SAMMNS(ph)PK_
H9JMY4	1.44	Up	0.0184341	S	DNA ligase 1-like	_VEPES(ph)DIELK_
kinases						
H9JQ06	1.39	Up	0.0448089	S	probable serine/threonine-protein kinase kinX	_NNPKPTS(ph)PPSSSK_
H9IU49	3.53	Up	0.0325598	S	protein kinase C	_GFS(ph)FVNK_
H9ISA9	1.34	Up	0.0038934	T	MAP/microtubule affinity-regulating kinase 3-like isoform X3	_QNT(ph)IDSASIK

Four classes of notable modulated proteins

Corrected *p*-value <0.05, fold change >1.3 or <0.77

**Table 2:** The phosphorylated proteins of BmNPV

Protein accession	24/N Ratio	Regulated	24/N *P* value	Amino acid	Protein names	Modified sequence
Viruse_ACQ57207.1	2.503	Up	2.02E-01	S	DBP	_KIGDSSS(ph)DDNQPK_
Viruse_ACQ57210.1	5.180	Up	1.37E-02	S	LEF-6	_RS(ph)LDS(ph)PR_
Viruse_ACQ57210.1	5.807	Up	4.73E-03	S	LEF-6	_RS(ph)LDS(ph)PR_
Viruse_ACQ57210.1	5.923	Up	6.13E-04	S	LEF-6	_LDGYVLASS(ph)PIPHTDWNEELK_
Viruse_ACQ57210.1	4.007	Up	2.99E-03	S	LEF-6	_LYAQSHGYDDDDDDLEDGEIDERDS(ph)LK_
Viruse_ACQ57210.1	4.040	Up	8.16E-04	S	LEF-6	_S(ph)LNNHLNDLNVLEK_
Viruse_ACQ57218.1	3.967	Up	3.92E-03	S	39K protein	_STS(ph)SSSSDNAAIPASK_
Viruse_ACQ57218.1	16.683	Up	7.26E-03	S	39K protein	_S(ph)TSSS(ph)SSDNAAIPASK_
Viruse_ACQ57218.1	2.287	Up	1.89E-02	S	39K protein	_STSSSS(ph)SDNAAIPASK_
Viruse_ACQ57218.1	1.407	Up	6.40E-03	S	39K protein	_NTTAAPTLLM(ox)VSDNTQDTNM(ox)S(ph)E_
Viruse_ACQ57239.1	3.773	Up	6.00E-03	S	Ac58-like protein	_AAYEIVRDDIDES(ph)DDNADNSTK_
Viruse_ACQ57239.1	1.693	Up	4.23E-02	T	Ac58-like protein	_QHTNTPPHYDT(ph)SEDEDEDNYYNY_
Viruse_ACQ57239.1	2.013	Up	2.60E-02	S	Ac58-like protein	_QHTNTPPHYDT(ph)SEDEDEDNY(ph)YNY_
Viruse_ACQ57239.1	2.013	Up	2.60E-02	Y	Ac58-like protein	_QHTNTPPHYDT(ph)SEDEDEDNY(ph)YNY_
Viruse_ACQ57247.1	3.827	Up	7.23E-03	S	LEF-3	_FFS(ph)GESSGEPLIK_
Viruse_ACQ57247.1	5.420	Up	5.59E-03	S	LEF-3	_MAMANS(ph)PKK_
Viruse_ACQ57261.1	3.033	Up	1.06E-02	S	Ac82-like protein	_VAS(ph)SPQLR_
Viruse_ACQ57261.1	3.690	Up	3.12E-03	T	Ac82-like protein	_TDAAVNT(ph)SSPKR_
Viruse_ACQ57261.1	4.527	Up	1.64E-02	S	Ac82-like protein	_TDAAVNTSS(ph)PK_
Viruse_ACQ57261.1	2.003	Up	4.04E-02	T	Ac82-like protein	_AVET(ph)ENDDDDDEDDAAS(ph)AIDEQK_
Viruse_ACQ57261.1	8.710	Up	1.62E-03	S	Ac82-like protein	_AVET(ph)ENDDDDDEDDAAS(ph)AIDEQK_
Viruse_ACQ57271.1	2.673	Up	2.81E-02	S	ODV-E25	_S(ph)PNAASTSSNVTMTR_
Viruse_ACQ57277.1	2.733	Up	5.64E-02	S	P6.9	_RS(ph)S(ph)TGATYGLTR_
Viruse_ACQ57277.1	2.073	Up	3.98E-02	S	P6.9	_RSS(ph)TGATYGLTR_
Viruse_ACQ57281.1	1.815	Up	4.22E-01	S	VP80	_LPINFLDTSATS(ph)PAVR_
Viruse_ACQ57313.1	3.340	Up	4.83E-03	S	ME53	_VKS(ph)LPTPVANSPLSPVR_
Viruse_ACQ57328.1	2.570	Up	4.66E-03	S	BRO-D	_S(ph)ISFDSLEEAQQFENR_
Viruse_ACQ57328.1	3.443	Up	1.11E-03	S	BRO-D	_SIS(ph)FDS(ph)LEEAQQFENR_
Viruse_ACQ57328.1	2.900	Up	5.71E-03	S	BRO-D	_SIS(ph)FDS(ph)LEEAQQFENR_

Corrected *p*-value <0.05, fold change >1.3

### Bioinformatics analysis

**Fig. 2:**
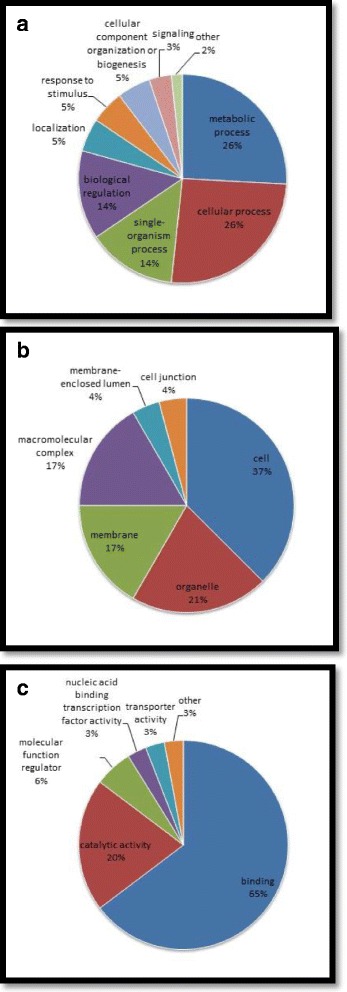
Pie chart of the distribution of phosphorylated proteins from gene ontology functional classification in terms of **a** biological processes, **b** cellular components, and **c** molecular functions

The GO annotation classified either the up-regulated or down-regulated proteins into three categories: biological processes, cellular components, and molecular functions. The up-regulated proteins in the biological process category were mainly identified at metabolic (9 proteins) and cellular processes (8 proteins); the down-regulated proteins were also mostly identified at cellular (7 proteins) and metabolic processes (6 proteins). The pie chart of the modulated proteins with respect to the biological processes, either the up- or down-regulated protein (Fig. 2a), showed that proteins distributed in metabolic and cellular processes have the same percentage (26%). Regarding cellular components, the up-regulated proteins were highly identified at cell (7 proteins) while the down-regulated proteins were apparently distributed evenly in the three components: membrane, organelle, and cell (2 proteins in each). Altogether, the distribution of up- or down-regulated proteins in the term of cellular component was mostly identified in the cell at 37% (Fig. 2b). Moreover, the most abundant modulated proteins in terms of molecular function were distributed in the binding activity: 32 up-regulated and 12down-regulated proteins, which amounted to 65% (Fig. 2c), the highest among the three main categories. These results strongly suggested that the binding activity in silkworm cell after BmNPV infection may be comprehensively regulated by phosphorylation. In addition, phosphorylation also seemed to exhibit an essential regulatory role in the cellular and metabolic processes.

**Fig. 3:**
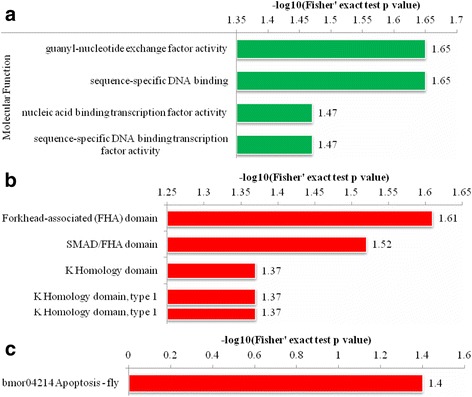
Functional enrichment analysis of the phosphorylated proteins in silkworm cells after BmNPV infection. All phosphorylated proteins in each group are over represented. *Red* and *green bars* are up-regulated and down-regulated proteins, respectively. **a** Enrichment analysis based on GO annotation. **b** Enrichment analysis based on domain annotation. **c** Enrichment analysis based on KEGG pathway annotation

**Fig. 4:**
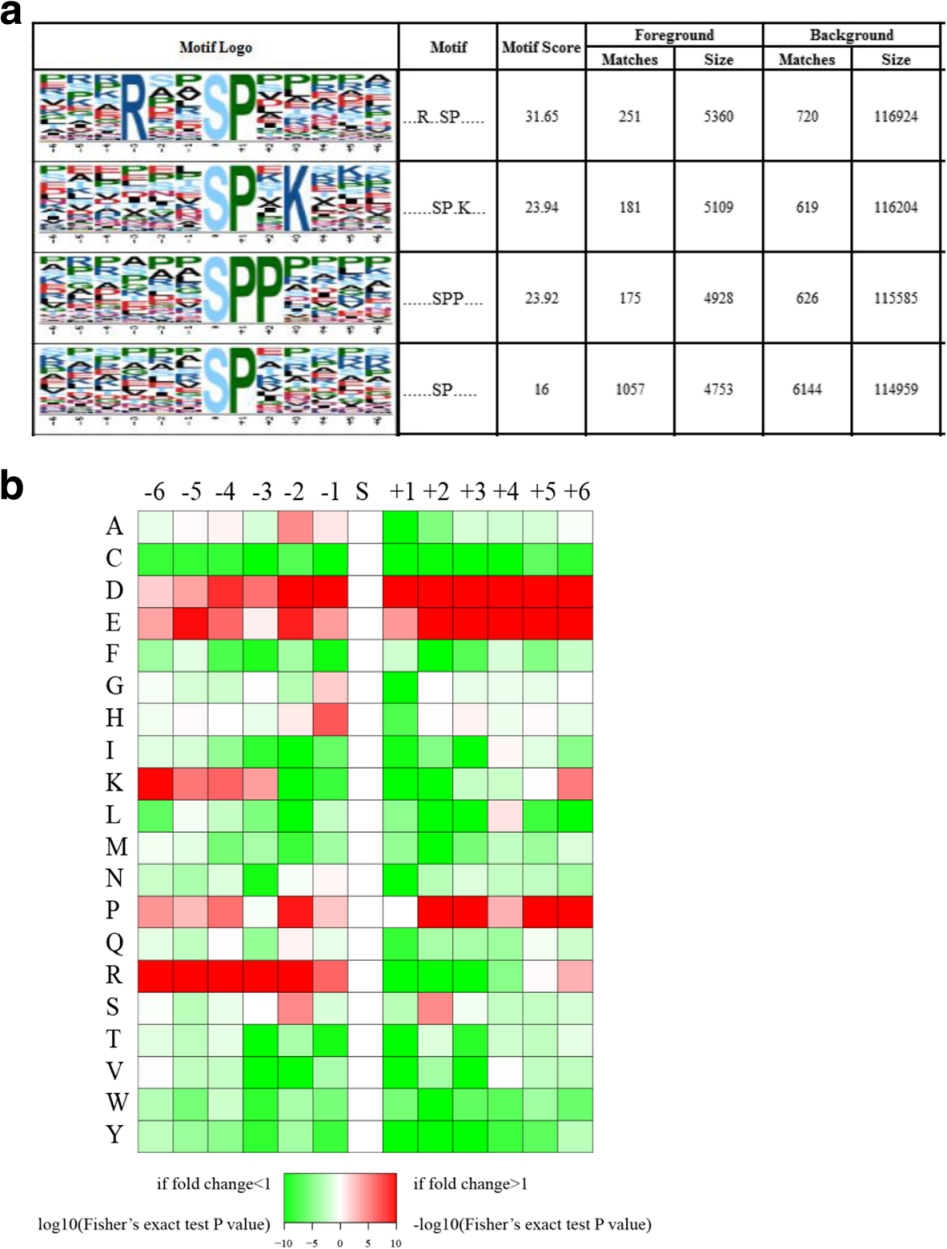
Motif analysis **a**. Phosphorylation motifs and sequence probability logos of remarkably modulated phosphorylation site motifs for ±6 amino acids around the phosphorylation sites. Motif score is obtained based on residues proximal to the fixed residues using the motif’s Position Weight Matrix (PWM). Background size indicates to the number of peptides identified and background matches indicate to the number of peptides containing the specific motif. Foreground size indicates to the number of phosphorylated peptides identified and foreground matches indicate to the number of phosphorylated peptides containing the specific motif. **b** The amino acid compositions heat map of phosphorylated sites representing the frequency of different kinds of amino acids around the phosphorylated sites. The *colors* represent the log_10_ of the ratio of amino acid frequency within 13 residues from the pS (phosphorylated Serine) residue versus S, *red* shows enrichment and *green* shows depletion

The functional enrichment was carried out in three types of analysis, i.e., gene ontology, domain, and KEGG pathway analysis. The GO enrichment analysis showed four modulated molecular functions listed in Fig. 3a, which indicated that most of them were involved in the binding activity. The four domains were reported to be up-regulated by domain enrichment analysis (Fig. 3b) while only bmor04214 apoptosis-fly pathway in the silkworm cells was enriched in the KEGG pathway analysis (Fig. 3c). The subcellular localization classification revealed that phosphorylated proteins in the treated *B. mori* cells compared with the control group were primarily located in the nucleus: either up-regulated (40 proteins) or down-regulated proteins (13 proteins). In addition, the cytosolic phosphorylated proteins were also highly identified in both up-regulated (13 proteins) and down-regulated (6 proteins) phosphoproteome (Table 3). The motif analysis was carried out to identify the over represented residues which are amino acids that often found both upstream and downstream of a modification site. Using soft motif-x resulting in four distinct phosphorylation motifs (Fig. 4), the pattern of amino acids distribution was observed from position −6 to +6 around the phosphorylation sites. We found that the hydrophobic amino acid, proline, was drastically over represented at position +1 following the phosphorylated serine at each of the six patterns. Moreover, proline was also enriched at +2 position in motif 3 indicating that the serine-rich region and hydrophobic amino acid (proline) region of the protein were preferred for phosphorylation. In addition, some basic amino acid was also over represented, i.e., arginine in −3 position at motif 1 and lysine in +3 position at motif 2 implying that polar, hydrophobic, and positively charged amino acids may be functionally critical for phosphorylation.

**Table 3:** The subcellular location classification of up and down-regulated proteins

Subcellular Location		No. of Protein
up-regulated	plasma membrane	5
	Cytosol	13
	Nuclear	40
	cytosol, nuclear	2
	Extracellular	3
	Peroxisome	1
	Mitochondria	2
Down-regulated	Nuclear	13
	plasma membrane	2
	Cytosol	6
	cytosol, nuclear	1
	Extracellular	1
	Mitochondria	1

## Discussion

The comprehensive phosphoproteome quantification has been extensively studied in several species attributable to its pivotal role leading to the altered gene expression through conformational changes in some functional proteins. *B. mori* and BmNPV were a topic of intense focus among several groups due to their correlation in the interaction of insect host and baculovirus pathogen with respect to the mechanism of virus invasion of the host [16] and the corresponding host immune system responses [17]. The present study analyzed the impact of infection of BmNPV to the global pattern of proteomic phosphorylation in silkworm cells. However, to our knowledge, this is the first study determining the phosphoproteome of *B. mori* after BmNPV infection resulting in a significant amount of candidate target phosphorylated proteins for deeper insight into the mechanism of phosphorylation to control the alterations on the host cells during viral infection.

Overall, the results showed that BmNPV infection greatly influenced the phosphorylation of diverse proteins related to several functions (Table 1). The notable alterations on the phosphorylation of proteins were involved in binding activity, protein synthesis, viral replication, and apoptosis through kinase activity and are further discussed below.

### Phosphorylation may have a pivotal function on the regulation of binding activity

As shown by the GO annotation, the most significant altered phosphoproteins (65%) were found in the binding activity for molecular function, either up-regulated or down-regulated (Fig. 2c). Among those modulated proteins, 34% were involved in nucleic acid binding; 10 proteins were up-regulated and 5 proteins were down-regulated. Although the phosphorylation ratio was little, more than 1/3 of the total number of proteins in the binding activity were involved in nucleic acid binding activity, indicating that phosphorylation may have a significant role in either the DNA or RNA binding activity in the silkworm cells after BmNPV infection. For instance, DNA topoisomerase II, one of the up-regulated proteins with 1.43 ratio phosphorylation, is a well-known enzyme in the process of transcription, replication, chromatin remodeling, and segregation [18]. This enzyme has been reported to have the ability to simplify the DNA topology by relaxing supercoiled DNA and unlinking knotted or catenated DNA which is in accordance with its physiological role in DNA replication and chromosome segregation [19, 20]. Moreover, the enzyme is also a predominant component of the nuclear scaffold and matrix [18, 21]. The matrix associated with the DNA regions was also reported to consist of topoisomerase II binding sites [22]. According to a search of the BmNPV genome sequence, it does not encode an ortholog of topoisomerase II. Furthermore, a recent report has proposed a plausible hypothesis that the intertwined baculovirus DNA was decatenated by topoisomerase 2 during viral replication [23]. Hence we hypothesize that the virus employs this enzyme for its genome replication by activating the enzyme through up-regulation. Study about topoisomerase for baculovirus is still lack. Further studies are necessitated to substantiate our hypothesis.

Another remarkable protein is the subunitssp27, a component of the upstream activation factor (UAF) complex, which interestingly has two phosphorylation sites in the position 84th and 88th of amino acid sequence with the elevation ratio 0.57- and 0.73-fold, respectively. Although in *B. mori* this protein is not yet examined, in yeast *Saccharomyces cerevisiae,* the upstream activation factor (UAF) is well-known as a multi-protein complex that tightly binds to the upstream element of the rDNA promoter and also strongly stimulates the transcription of RNA polymerase I (Pol I) [24]. Subunit spp27 of this protein is a homolog from *Schizosaccharomyces pombe* which has UAF-like activity [25] such as the ability to interact with rDNA upstream sequence binding protein; Acr1, an accumulation of condensing at rDNA 1 which has been reported to be weakly similar to RRN9, a component of *Saccharomyces cereviseae* UAF complex [26]. Subunit spp27 also has been reported to have good alignment with UAF30p, another subunit of UAF complex of *S. cereviseae* [25]. Furthermore, UAF also plays a critical role in the silencing of transcription through RNA polymerase II (Pol II) by stabilizing Pol I state [24]. The down-regulation of this protein may cause the inhibition of the silencing, which leads to the activation of transcription by Pol II. This activation of Pol II might correspond with the BmNPV-based manipulation of the host gene by miRNA that is canonically transcribed by Pol II. By encoding a miRNA, the BmNPV represses the expression of the silkworm GTP-binding nuclear protein Ran, resulting in the decrease of the host small-RNA population [27]. The down-regulation of UAF causing all those possible consequents which are eventually leading to the alleviation of the amount of host small-RNA might be required for the enhancement of BmNPV load in the infected cells.

Another intriguing result is that thymosin isoform XI (tiXI) is an up-regulated protein with four sites exhibiting high phosphorylation ratio. Those four sites were located at position 106th, 30th, 31st, and 107th on the amino acid sequence and the phosphorylation elevation ratios compared with the control group were 1.99, 2.0, 2.9, and 1.82, respectively. Although tiXI is not widely focused, the family of this protein, thymosin beta 4 (Tβ4), has been extensively studied in several species and is known to have a pivotal role in the organization of cytoskeleton by binding or sequestering to actin monomers [28]. This protein regulates the actin cytoskeleton dynamics through retaining a large pool of actin monomers which interact with globular actin to produce a 1:1 complex for controlling the filamentous actin assembly [29]. Tβ4 regulates the equilibrium between the globular and filamentous actin, which is vital for rapid rearrangement of the cytoskeleton [30]. The hyperphosphorylation of this protein after BmNPV infection might activate the cellular Tβ4 protein which is responsible to the filamentous actin to globular actin ratio alteration [31]. This regulation appears close relation with the infection process of BmNPV. The actin and microtubule have significant roles in every virus life cycle. Viruses have ability to reorganize the cytoskeleton and restructure the host transport equipment to fulfill their needs [32], for instance, H5N1 could manipulate and exploit diverse host cytoskeletal protein to promote their infection [33]. AcMNPV has been reported to harness actin polymerization based motility in two infection phases. In the initial phase right after cell entry, they utilize motility for cytoplasm exploration and translocation to the nucleus through nuclear periphery for initiation the viral gene. In the next phase, after early gene expression, AcMNPV required motility to accumulate the distinct set of nucleocapsid at the tips of actin-rich surface spikes [34]. The up-regulation occurred on four sites of this tiXI protein with a high phosphorylation ratio after BmNPV infection strongly suggested that phosphorylation might have a predominant influence on cytoskeleton change through actin binding which is important for life cycle of every virus. Further studies are highly recommended to authenticate our hypothesis.

### Phosphorylation may represent an obligatory regulation in protein synthesis

Similar to other viruses, baculoviruses employ the host chaperones to assist the rapid synthesis of a large quantity of their viral proteins [35]. Baculoviruses completely rely on the host translational equipment such as ribosomes, tRNA, amino acid metabolism and transport, chaperones for protein folding and other translation factors. The presence of AT-rich regions and unstructured 5′ untranslated (UTR) regions of the virus mRNA allow the baculovirus to compete with host mRNA for translation by its higher binding activity to host translation factor [36]. A previous study revealed some translational factors in silkworm cells that were commonly up-regulated after BmNPV infection such as eukaryotic initiation factor (eIF3–6, eIF1A, aIF3-2b) and an elongation factor (EF1d) [16] corresponding to the present study, resulting in the hyperphosphorylation of EF1d with fold elevation ratio 1.69. Many studies have reported that the EF1d phosphorylation could alter translational efficiency [37–39]. Furthermore, in mammalian cell infected by herpes viruses, EF1d also phosphorylated by conserved protein kinases encoded by these viruses suggesting that it plays an important role of efficient replication of these viruses [40]. The up-regulation of BmNPV EF1d suggested that this virus might employ EF1d and enhance its translational efficiency for viral replication through phosphorylation.

Another notably up-regulated phosphoprotein is ATP-dependent RNA helicase DDX10. RNA helicase is well-known as an essential factor in most of the RNA metabolism processes including ribosome biogenesis, pre-mRNA splicing and translation initiation [41, 42]. DDX10 is categorized in the group of DEAD-box family, the largest group of helicases [43]. Although this protein from silkworm is yet less studied, the other proteins in the family from different species have been elucidated for the correlation with the viral infection. DDX1 is the common helicase from DEAD-box family, which has been reported as a cellular co-factor for viral replication by several viruses such as coronavirus [44], JC virus [45], hepatitis C virus [46], and also HIV-1 for replication and nuclear export [47]. With a high fold elevation ratio (2.31), the hyperphosphorylation of ATP-dependent RNA helicase DDX10 protein is designated to play a major role in protein synthesis in silkworm cells after BmNPV infection. Thus, further studies are imperative to better understand the role of hyperphosphorylation in the ATP-dependent RNA helicase DDX10 for protein synthesis of *B. mori* cells after BmNPV infection.

### Phosphorylation may be responsible for viral replication during infection

During infection, while the host DNA replication is detained, viral replication is rapidly conducted harnessing the host replication machinery and concentrating in a virogenic stroma, which is a separate compartment within the nucleus [48, 49]. DNA ligase 1-like with the fold elevation ratio 1.44, is one of the up-regulated proteins that is associated with the replication of DNA. DNA ligase has been highlighted as a crucial factor in the linkage of Okazaki fragment on the lagging strand [50]. Interestingly, the Viral DNA ligase of vaccinia virus has been reported to harness cellular Topoisomerase 2 to site the viral replication and assembly [51]. However, BmNPV apparently does not encode DNA ligase, only some baculoviruses do, such as LdMNPV (*Lymantria dispar* multicapsid nuclear polyhedrosis virus), according to the BLAST result, its DNA ligase is most optimally aligned with the *B. mori* DNA ligase 3 isoform X1 after other lepidopteran suggesting that the DNA ligase of LdMNPV was probably acquired from host. The fact that BmNPV genome does not have the ortholog of DNA ligase suggested that the up-regulation of cellular DNA ligase may be important either for viral replication or assembly.

The origin recognition complex subunit 2 (orc2), with a high phosphorylation ratio (5.69), is the most hyperphosphorylated protein for DNA replication and the highest up-regulated protein among all the different classes of proteins. Although the mechanism in detail remains elusive, the viral replication of BmNPV has been reported to affect the expression of *BmORCs* [52]*.* In the case of human, ORC is known as important protein which is able to initiate DNA replication by facilitating the establishment of the pre-replication complex (pre-RC) at the origin of DNA replication [53]. ORC is also known to be associated with an EBV (Epstein-Barr virus) replication origin for the propagation of its genome by utilizing antibodies against the three different subunits of human ORC in order to precipitate the cross-linked chromatin [54]. The up-regulation of *B. mori* orc2 protein after BmNPV infection was probably important for the activation of this protein. Together with DNA ligase, we speculated that they have an essential function for the viral genome replication of BmNPV. Further research is highly recommended to ascertain this hypothesis.

### Phosphorylation may regulate anti-apoptotic cells through the kinases activity after BmNPV infection

Apoptosis is a genetically programmed cell death as an emergency response to a variety of stimuli such as radiation damage, aberrant growth or viral infection [55]. Some viruses such as Oropouche virus (OROV) induce apoptosis in the cells right after their infection that causes degeneration in cells shortly after infection [56]. However, some viruses evolve strategies to evade early apoptosis in order to avoid the elimination of progeny virus spread and the limitation of virus production [55]. For instance, an inhibitor of apoptosis proteins (IAPs), a family of proteins which were originally discovered in baculovirus has been reported to be involved in suppressing the host cell death in response to viral infection [57, 58]. Protein kinases C (PKC) family members are associated with an extensive range of cellular responses including cell permeability, migration, hypertrophy, proliferation, secretion, and also apoptosis. In particular, PKC cascades are apparently essential modulators of the apoptotic response [59]. A recent study showed a high up-regulated phosphorylation of PKC in the silkworm cells after BmNPV infection. The hyperphosphorylated PKC occurred in the epsilon isoform (PKCε). Among several members of PKC protein family, some isoforms induceapoptosis, and the others inhibit it. PKCε possesses the ability to enhance tumorigenesis that can associate with the suppression of apoptosis [60]. PKCε has also been reported to trigger cellular survival by initiating the NF-κB pathway [61], which leads to the activation of gene transcription encoding IAPs [62]. With PKCε high phosphorylation ratio (3.53), we strongly suggested that the hyperphosphorylation of this protein has a great implication on the survival of host cells during BmNPV infection.

### The phosphorylation of BmNPV protein

All of the quantified proteins of BmNPV are up-regulated; some of them have a notable phosphorylation ratio and are even at more than one site. 39 K protein, also known as Bmorf 27, is one of the prominent hyperphosphorylated proteins of BmNPV which has 4 phosphorylated sites and one of them has the highest phosphorylation ratio among all of BmNPV phosphorylated protein and is even higher than it of *B. mori* phosphorylated protein. It has been well known that 39 K promoters have important utility for insect cell engineering such as BEV system by providing the highest level of tightly regulated protein production [63]. It also has been reported that BmNPV 39 K plays an important role in viral late gene transcription. Moreover, the deletion of this gene resulted in very poor budded virus production, low polyhedral production, oral infectivity attenuation, and deprived virogenic stroma formation [64]. The homolog of 39 k, AcMNPV orf 36 also called as pp31 has been reported to be phosphorylated as well [65]. Although the function of pp31 AcMNPV phosphorylation remains elusive, the great phosphorylation ratio of 39 K phosphorylation strongly suggested that phosphorylation may have important role for this protein in promoting protein production. Further study is necessary for supporting this hypothesis.

BmNPV protamine-like protein named P6.9 underwent phosphorylation during infection; it has two phosphorylated sites with phosphorylation ratio of 2.73 and 2.07, respectively. Although in BmNPV this protein has not been well assessed but in *Autographa californica* multiple nucleopolyhedrovirus (AcMNPV), P6.9 has been reported to be transiently phosphorylated in infected cells prior to nucleocapsid assembly [66]. This protein binds and condenses baculoviral DNA for packing into capsids [67]. By the time nucleocapsids enter nuclei through nuclear pores, P6.9 is phosphorylated which could repel the negatively charged DNA, leading to the viral genome release associated with capsid and kinase [68, 69]. A recent study indicated that during infection there are 13 Ser/Thr phosphorylation sites on P6.9 AcNMPV which interestingly 7 of those are reported to be dependent to PK1 (protein kinase 1). Moreover, replacement of those 7 Ser/Thr phosphorylated residues with Ala in P6.9 AcNMPV significantly diminishes the transcription of the very late viral genes and viral infectivity [70]. Further research is necessary to find whether phosphorylation of P6.9 BmNPV has an important role on DNA binding activity and if PK1 also has influence to the phosphorylation of this protein.

Another remarkable protein of BmNPV is LEF-6 with 5 phosphorylated sites; the phosphorylation ratio in every site was relatively high. In AcMNPV lef-6 has been reported to show ability in accelerating the viral lifecycle and increasing virus yields [71]. The study on this protein phosphorylation is still lacking. However, considering its 5 hyperphosphorylated sites, we strongly suggest that phosphorylation on this protein may play significant role in the viral yielding and lifecycle.

## Conclusions

The current study comprehensively identified the global pattern of phosphorylation of *B. mori* after BmNPV infection, harnessing a highly sensitive proteomic method. In total, 4764 phosphorylation sites in 1717 proteins were quantified in silkworm cells, which were extensively involved in the regulation of several aspects of metabolism and molecular function during BmNPV infection. These results also suggest a large number of candidate target phosphorylated proteins for in-depth studies on the mechanism of phosphorylation modulating the alterations on the host cells during viral infection. Furthermore, we classified and discussed most of the differentially and remarkably phosphorylated proteins into several functional groups, i.e., binding activity, protein synthesis, viral replication, and apoptosis through kinase activity. There are also remarkable proteins of BmNPV which were modified during infectionsuch as P6.9, 39 K, LEF-6, Ac58-like protein, Ac82-like protein, and BRO-D among which most of those sites have relative high phosphorylation ratio and have more than one hyperphosphorylated site. Nevertheless, there will be undeniable cross-talk between these groups despite the phosphorylated proteins are involved in multiple molecular functions and cellular processes.

## Abbreviations

AcMNPV: *Autographa californica* multiple nucleopolyhedrovirus
AGC: Automatic gain control
BEVS: Baculovirus expression vector system
BmNPV: *B. mori* nucleopolyhedrovirus
DBP: DNA binding protein
DTT: Dithiothreitol
EBV: Epstein-Barr virus
EF: Elongation factor
eIF: Eukaryotic initiation factor
FDR: The false discovery rate
GO: Gene ontology
IAA: Iodoaceta amide
IAPs: Inhibitor of apoptosis proteins
Kac: Lysine acetylation
KEGG: Kyoto encyclopedia of genes and genome
LdMNPV: *Lymantria dispar* multicapsid nuclear polyhedrosis virus
MOI: Multiplicity of infection
ORC2: The origin recognition complex subunit 2
OROV: Oropouche virus
PKCε: Protein kinase C epsilon
Pol I: RNA polymerase I
Pol II: RNA polymerase II
pre-RC: pre-Replication complex
PTM: Post-translational modification
TCA: Trichloro acetic acid
TEAB: Triethyl ammonium bicarbonate
tiXI: Thymosin isoform XI
TMT: Tandem mass tag
UAF: Upstream activation factor
UTR: Untranslated region

## References

[1] Xia Q, Zhou Z, Lu C, Cheng D, Dai F, Li B (2004). A draft sequence for the genome of the domesticated silkworm (*Bombyx mori*). Science.

[2] Jiang L, Xia Q (2014). The progress and future of enhanching antiviral capacity by transgenic technology in the silkworm *Bombyx mori*. Insect Biochem Mol Biol Rev.

[3] Sekimizu N, Paudel A, Hamamoto H (2012). Animal welfare and use of silkworm as a model animal. Drug Discov Ther.

[4] Mita K (2009). Genome of a lepidopteran model insect, the silkworm *Bombyx mori*. Seikagaku.

[5] Mon H, Izumi M, Mitsunobu H, Tatsuke T, Liyama K, Jikuya H (2011). Post-translational modification of the N-terminal tail of histone H3 in holocentric chromosomes of *Bombyx mori*. Insect Biochem Mol Biol.

[6] Nie Z, Zhu H, Zhou Y, Wu C, Liu Y, Sheng Q (2015). Comprehensive profiling of lysine acetylation suggest the widespread function is regulated by protein acetylation in the silkworm, *Bombyx mori*. Proteomics.

[7] Jiang L, Zhao P, Cheng T, Sun Q, Peng Z, Dang Y (2013). A transgenic animal with antiviral properties that might inhibit multiple stages of infection. Antivir Res.

[8] Lee TI, Young RA (2000). Transcription of eukaryotic protein-coding genes. Annu Rev Genet.

[9] Lee DF, Chen CC, Hsu TA, Juang JL (2000). A baculovirus super infection system: efficient vehicle for gene transfer into drosophila S2 cells. J Virol.

[10] Deribe YL, Pawson T, Dikic I (2010). Post-translational modifications in signal integration. Nat Struct Mol Biol.

[11] Chang C, Stewart RC (1998). The two-component system. Regulation of diverse signaling pathways in prokaryotes and eukaryotes. Plant Physiol.

[12] van Weeren PC, de Bruyn KM, de Vries-Smits AM, van Lint J, Burgering BM (1998). Essential role for protein kinase B (PKB) in insulin-induced glycogen synthase kinase 3 inactivation. Characterization of dominant-negative mutant of PKB. J Biol Chem.

[13] Cole PA, Shen K, Qiao Y, Wang D (2003). Protein tyrosine kinases Src and Csk: a tail's tale. Curr Opin Chem Biol.

[14] Babior BM (1999). NADPH oxidase: an update. Blood.

[15] Zemskov EA, Kang W, Maeda S (2000). Evidence for nucleic acid binding ability and nucleosome association of *Bombyx mori* nucleopolyhedrovirus BRO proteins. J Virol.

[16] Rahman MM, Gopinathan KP (2004). Systemic and in vitro infection process of *Bombyx mori* nucleopolyhedrovirus. Virus Res.

[17] Xue J, Qiao N, Zhang W, Cheng RL, Zhang XQ, Bao YY (2012). Dynamic interaction between *Bombyx mori* nucleopolyhedrovirus and its host cells revealed by transcriptome analysis. J Virol.

[18] Earnshaw WC, Heck MM (1985). Localization of topoisomerase II in mitotic chromosomes. J Cell Biol.

[19] Liu LF, Liu CC, Alberts BM (1980). Type II DNA topoisomerases: enzymes that can unknot a topologically knotted DNA molecule via a reversible double-strand break. Cell.

[20] Ullsperger CJ, Vologodskii AV, Cozzarelli NR, Eckstein F, David M, Lilley J (1995). Unlinking of DNA topoisomerase during DNA replication. Nucleic acid and molecular biology.

[21] Berrios M, Osheroff N, Fisher PA (1985). In situ localization of DNA topoisomerase II, a major polypeptide component of the drosophila nuclear matrix fraction. Proc Natl Acad Sci U S A.

[22] Wu Y, Wu Y, Wu Y, Tang H, Wu H, Zhang G (2013). Screening of candidate proteins interacting with IE-2 of BmNPV. Mol Biol Rep.

[23] Volkman LE (2015). Baculoviruses and nucleosome management. Virology.

[24] Vu L, Siddiqi I, Lee BS, Josaitis CA, Nomura M (1999). RNA polymerase switch in transcription of yeast rDNA: role of transcription factor UAF (upstream activation factor) in silencing rDNA transcription by RNA polymerase II. Proc Natl Acad Sci U S A.

[25] Liu M, Guo A, Boukhgalter B, Van Den Heuvel K, Tripp M (2002). Et el. Characterization of the fission yeast ribosomal DNA binding factor: components share homology with upstream activating factor and with SWI/SNF subunits. Nucleic Acids Res.

[26] Nakazawa N, Nakamura T, Kokubu A, Ebe M, Nagao K, Yanagida M (2008). Dissection of the essential steps for condensin accumulation at kinetochores and rDNAs during fission yeast mitosis. J Cell Biol.

[27] Singh CP, Singh J, Nagaraju J (2012). A baculovirus-encoded microRNA (miRNA) suppresses its host miRNA biogenesis by regulating the exportin-5 cofactor ran. J Virol.

[28] Sanger JM, Golla R, Safer D, Choi JK, Yu KR, Sanger JW (1995). Increasing intracellular concentrations of thymosin beta 4 in PtK2 cells: effects on stress fibers, cytokinesis, and cell spreading. Cell Motil Cytoskeleton.

[29] Safer D, Elzinga M, Nachmias VT (1991). Thymosin beta 4 and Fx, an actin-sequestering peptide, are indistinguishable. J Biol Chem.

[30] Grant DS, Kinsella JL, Kibbey MC, LaFlamme S, Burbelo PD, Goldstein AL (1995). Matrigel induces thymosin beta 4 gene in differentiating endothelial cells. J Cell Sci.

[31] Kim S, Kwon J (2015). Actin cytoskeletal rearrangement and dysfunction due to activation of the receptor for advanced glycation end products is inhibited by thymosin beta 4. J Physiol.

[32] Radtke K, Döhner K, Sodeik B (2006). Viral interactions with the cytoskeleton: a hitchhiker's guide to the cell. Cell Microbiol.

[33] Li Y, Ming F, Huang H, Guo K, Chen H, Jin M (2017). Proteome response of chicken embryo fibroblast cells to recombinant H5N1 avian influenza viruses with different neuraminidase stalk lengths. Sci Rep.

[34] Ohkawa T, Volkman LE, Welch MD (2010). Actin-based motility drives baculovirus transit to the nucleus and cell surface. J Cell Biol.

[35] Champoux JJ (2001). DNA topoisomerases: structure, function, and mechanism. Annu Rev Biochem.

[36] Scheper GC, Vries RG, Broere M, Usmany M, Voorma HO, Vlak JM (1997). Translational properties of the untranslated regions of the p10 messenger RNA of *Autographa californica* multicapsid nucleopolyhedrovirus. J Gen Virol.

[37] Chang YW, Traugh JA (1997). Phosphorylation of elongation factor 1 and ribosomal protein S6 by multipotential S6 kinase and insulin stimulation of translational elongation. J Biol Chem.

[38] Minella O, Cormier P, Morales J, Poulhe R, Belle R, Mulner-Lorillon O (1994). cdc2 kinase sets a memory phosphorylation signal on elongation factor EF-1 delta during meiotic cell division, which perdures in early development. Cell Mol Biol (Noisy-Le-Grand).

[39] Venema RC, Peters HI, Traugh JA (1991). Phosphorylation of elongation factor 1 (EF-1) and valyl-tRNA synthetase by protein kinase C and stimulation of EF-1 activity. J Biol Chem.

[40] Kawaguchi Y, Kato K, Tanaka M, Kanamori M, Nishiyama Y, Yamanashi Y (2003). Conserved protein Kinases encoded by Herpesviruses and cellular protein Kinase cdc2 target the same Phosphorylation site in eukaryotic elongation factor 1δ. J Virol.

[41] Jankowsky E (2010). RNA Helicases at work: binding and rearranging. Trends Biochem Sci.

[42] Fairman-Williams ME, Guenther UP, Jankowsky E (2010). SF1 and SF2 helicases: family matters. Curr Opin Struct Biol.

[43] Gorbalenya AE, Koonin EV (1993). Helicases: amino acid comparisons and structure-function relationships. Curr Opin Struct Biol.

[44] Xu L, Khadijah S, Fang S, Wang L, Tay FP, Liu DX (2010). The cellular RNA helicase DDX1 interacts with coronavirus nonstructural protein 14 and enhances viral replication. J Virol.

[45] Sunden Y, Semba S, Suzuki T, Okada Y, Orba Y, Nagashima K (2007). Identification of DDX1 as a JC virus transcriptional control region-binding protein. Microbiol Immunol.

[46] Pan TT, Fang CY, Yi ZG, Yang PY, Yuan ZG (2006). Subproteomic analysis of the cellular proteins associated with the 3′ untranslated region of the hepatitis C virus genome in human liver cells. Biochem Biophys Res Commun.

[47] Edgcomb SP, Carmel AB, Naji S, Ambrus-Aikelin G, Reyes JR, Saphire AC (2012). DDX1 is an RNA-dependent ATPase involved in HIV-1 rev function and virus replication. J Mol Biol.

[48] Braunagel SC, Parr R, Belyavskyi M, Summers MD (1998). *Autographa californica* Nucleopolyhedrovirus infection results in Sf9 cell cycle arrest at G2/M phase. Virology.

[49] Vanarsdall AL, Okano K, Rohrmann GF (2005). Characterization of the replication of a baculovirus mutant lacking the DNA polymerase gene. Virology.

[50] Nguyen Q, Nielsen LK, Reid S (2013). Genome scale transcriptomics of baculovirus-insect interactions. Viruses.

[51] Lin YCJ, Li J, Irwin CR, Jenkins H, DeLange L, Evans DH (2008). Vaccinia virus DNA ligase recruits cellular topoisomerase II to sites of viral replication and assembly. J Virol.

[52] Yang HP, Luo SJ, Li YN, Zhang YZ, Zhang ZF (2011). Identification and characterization of the DNA replication origin recognition complex gene family in the silkworm *Bombyx mori*. Biosci Rep.

[53] Shen Z, Prasanth SG (2012). Orc2 protects ORCA from ubiquitin-mediated degradation. Cell Cycle.

[54] Chaudhuri B, Xu H, Todorov I, Dutta A, Yates JL (2001). Human DNA replication initiation factors, ORC and MCM, associate with oriP of Epstein-Barr virus. Proc Natl Acad Sci U S A.

[55] Teodoro JG, Branton PE (1997). Regulation of apoptosis by viral gene products. J Virol.

[56] Acrani GO, Gomes R, Proença-Módena JL, da Silva AF, Carminati PO, Silva ML (2010). Apoptosis induced by Oropouche virus infection in HeLa cells is dependent on virus protein expression. Virus Res.

[57] Crook NE, Clem RJ, Miller LK (1993). An apoptosis-inhibiting baculovirus gene with a zinc finger-like motif. J Virol.

[58] RJ BMJC, Miller LK (1994). An apoptosis-inhibiting gene from a nuclear polyhedrosis virus encoding a polypeptide with Cys/his sequence motifs. J Virol.

[59] Utz P, Anderson P (2000). Life and death decisions: regulation of apoptosis by proteolysis of signaling molecules. Cell Death Differ.

[60] Wheeler DL, Martin KE, Ness KJ, Li Y, Dreckschmidt NE, Wartman M (2004). Protein kinase C epsilon is an endogenous photosensitizer that enhances ultraviolet radiation-induced cutaneous damage and development of squamous cell carcinomas1. Cancer Res.

[61] Catley MC, Cambridge LM, Nasuhara Y, Ito K, Chivers JE, Baton A (2004). Inhibitors of protein kinase C (PKC) prevent activated transcription: role of events downstream of NF-KappaB DNA binding. J Biol Chem.

[62] Danial NN, Korsmeyer SJ (2004). Cell death: critical control points. Cell.

[63] Lin CH, Jarvis DL (2013). Utility of temporally distinct baculovirus promoters for constitutive and baculovirus-inducible transgene expression in transformed insect cells. J Biotechnol.

[64] Gomi S, Zhou CE, Yih W, Majima K, Maeda S (1997). Deletion analysis of four of eighteen late gene expression factor gene homologues of the baculovirus, BmNPV. Virology.

[65] Broussard DR, Guarino LA, Jarvis DL (1996). Dynamic phosphorylation of *Autographa californica* nuclear polyhedrosis virus pp31. J Virol.

[66] Oppenheimer DI, Volkman LE (1995). Proteolysis of p6.9 induced by cytochalasin D in *Autographa californica* M nuclear polyhedrosis virus-infected cells. Virology.

[67] Tweeten KA, Bulla LA, Consigli RA (1980). Characterization of an extremely basic protein derived from granulosis virus nucleocapsids. J Virol.

[68] Funk CJ, Consigli RA (1993). Phosphate cycling on the basic protein of Plodia interpunctella granulosis virus. Virology.

[69] Wilson ME, Consigli RA (1985). Functions of a protein kinase activity associated with purified capsids of the granulosis virus infecting Plodia interpunctella. Virology.

[70] Li A, Zhao H, Lai Q, Huang Z, Yuan M, Yang K (2015). Posttranslational modifications of Baculovirus Protamine-like protein P6.9 and the significance of its hyperphosphorylation for viral very late Gene Hyperexpression. Sandri-Goldin RM ed. J Virol.

[71] Lin G, Blissard GW (2002). Analysis of an *Autographa californica* multicapsid nucleopolyhedrovirus LEF-6-null virus: LEF-6 is not essential for viral replication but appears to accelerate late gene transcription. J Virol.

